# Supplementary material for: Ultra-stable low-coordinated PtSA/CeZrO2 ordered macroporous structure integrated industrial-scale monolithic catalysts for high-temperature oxidation
Source: Nat Commun. 2025 Aug 22;16:7847. doi: 10.1038/s41467-025-63112-y (PMC12373748; doi:10.1038/s41467-025-63112-y)
Supplement: Supplementary file 1 — Supplementary Information [file 41467_2025_63112_MOESM1_ESM.pdf]

Supplementary Information

Ultra-stable Low-coordinated $Pt_{SA}/CeZrO_2$ Ordered Macroporous structure Integrated Industrial-scale Monolithic Catalysts for High-Temperature Oxidation

Baojian Zhang[1,2,3], Rui Liu[1,2,3], Liangwei Li[1,2,3], Weihong Guo[1,2,3], Biluan Zhang[1,2], Bosheng Chen[1,2], Weidong Yuan[1,2], Pan Li[1,2,3], Shaowen Zhang[1,2], Jinlong Wang[1,2,3], Ji Yang[1,2,3], Zhu Luo[1,2,3]*, Yanbing Guo[1,2,3]*

[1]Institute of Environmental and Applied Chemistry, College of Chemistry, Central China Normal University, Wuhan 430079, P. R. China

[2]Engineering Research Center of Photoenergy Utilization for Pollution Control and Carbon Reduction, Ministry of Education, Central China Normal University, Wuhan 430079, P. R. China

[3]Wuhan Institute of Photochemistry and Technology, Wuhan 430083, P. R. China

*E-mail: guoyanbing@mail.ccnu.edu.cn. luo.z@ccnu.edu.cn.

This Supplementary Information file includes: Page

Supplementary Method…………………………………………………………......3

Supplementary Figures 1-19…………………………………………………….……..4

Supplementary Tables 1-3………………………………………..………........37

Supplementary References………………………………………………….........40

Supplementary Method

Details about catalyst synthesis:

Chemical Materials. Cerium nitrate hexahydrate (Ce $(NO_3)_3 \cdot 6H_2O$), ethanol (C_2H_5OH), zirconyl chloride octahydrate ($ZrOCl_2 \cdot 8H_2O$), citric acid monohydrate ($C_6H_8O_7 \cdot H_2O$), Tetraammineplatinum nitrate ($Pt(NH_3)_4(NO_3)_2$). These chemicals were sourced from Sinopharm Chemical Reagent Co. Ltd. Additionally, polystyrene (PS), synthesized in-house, was utilized. Polyvinyl pyrrolidone (PVP) was acquired from Sigma-Aldrich and used as received, without further purification. The cordierite honeycomb ceramic substrate was procured from Aofu Inc, located in Shandong Province, China.

Dispersion of the PS sphere in $Ce_{0.8}Zr_{0.2}O_2$ precursor sol. First, dissolve 20 mmol of $Ce(NO_3)_3 \cdot 6H_2O$ and 5 mmol of $ZrOCl_2 \cdot 8H_2O$ in 50 ml of ethanol (C_2H_5OH) solution, and stir until uniform. Then add 25 mmol of citric acid ($C_6H_8O_7 \cdot H_2O$) and stir for 30 minutes to obtain a $Ce_{0.8}Zr_{0.2}O_2$ precursor sol. Next, add 25 ml of a 10% solid content PS (polystyrene) microsphere ethanol dispersion to the precursor sol and stir for 10 minutes.

Supplementary Figures

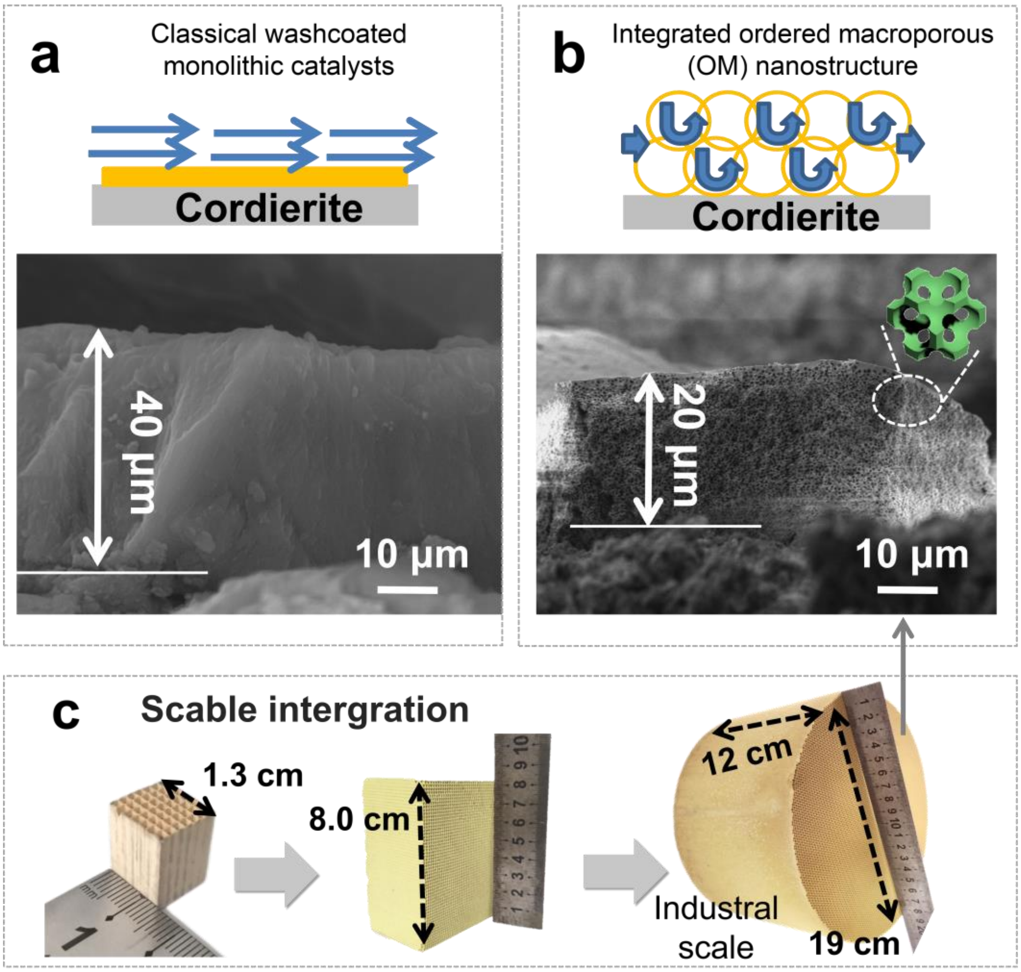

Supplementary Fig. 1: Scheme and cross view-SEM images of (**a**) traditional-washcoated monolithic-catalysts and (**b**) integrated ordered macroporous (OM) structure $Pt_{SA}/CeZrO_2$; (**c**) Photographs of monolithic OM $Pt_{SA}/CeZrO_2$ catalyst device with different size.

In **Supplementary Fig. 1a** and **b**, it can be seen that the traditional-washcoated monolithic catalysts have a dense layer with a thickness of 40 µm, while the new OM structure layer shows a clear porous structure with a coating thickness of 20 µm.

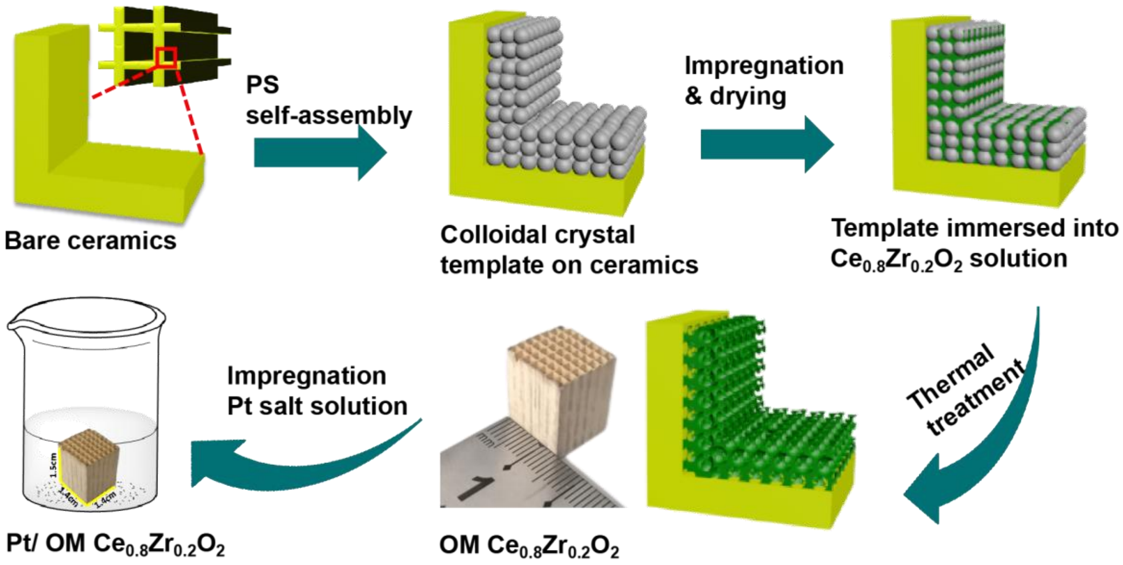

Supplementary Fig. 2: Fabrication process of Pt/OM $CeZrO_2$ (2.5 cm^3).

Supplementary Fig. 2 is a schematic diagram of the preparation method for small Pt/OM $CeZrO_2$ (2.5 cm^3). First, PS microspheres are coated onto the surface of the substrate, then the sol is impregnated. After that, template removal and metal oxide crystallization are achieved through drying and calcination steps. Finally, Pt is loaded using the impregnation method.

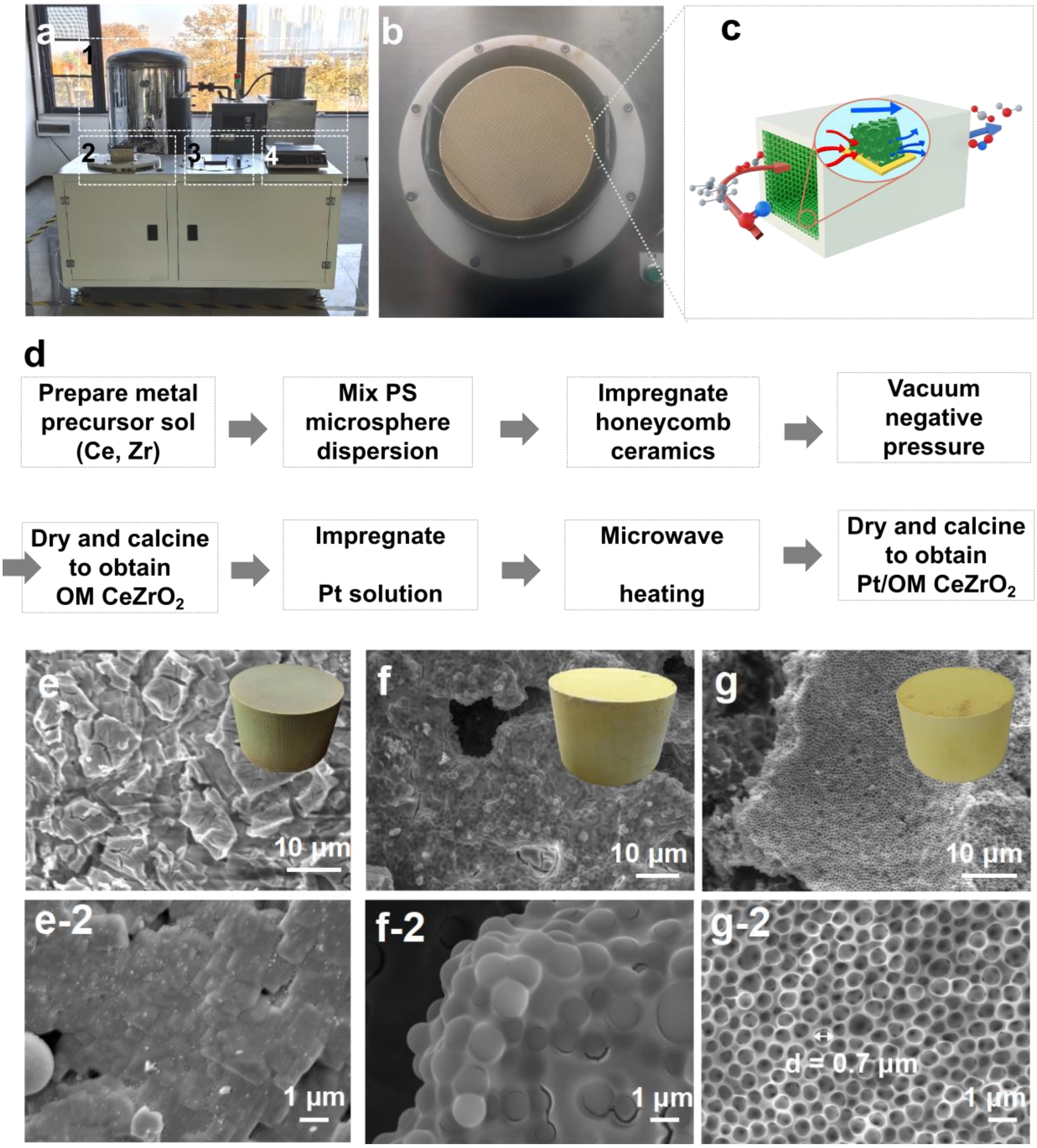

Supplementary Fig. 3: (**a**) Actual photo of the coating device; (**b**) Catalyst in the vacuum tank; (**c**) Schematic diagram of the OM catalyst; (**d**) Synthesis process of the OM catalyst; (**e**) SEM of the Powder catalyst surface; (**f**) SEM of the OM catalyst surface before calcination; (**g**) SEM of the OM catalyst surface after calcination and template removal.

Supplementary Fig. 3a shows the actual photo of the macro-scale preparation device for OM integral catalysts (3.4 L), where **label 1** indicates the buffer tank of the device, **label 2** indicates the vacuum drawing function area of the device, **label 3** indicates the impregnation

coating function area, and **label 4** indicates the weighing area. **Supplementary Fig. 3b** displays the state of a 3.4 L cylindrical catalyst in the vacuum tank. **Supplementary Fig. 3c** shows a microscopic illustration of a channel in the catalytic device**. Supplementary Fig. 3d** outlines the macro-scale preparation process of the OM catalyst, which is achieved through a simple templating and impregnation method. The impregnation is at **label 3**, and the vacuum negative pressure is at **label 2**. **Supplementary Fig. 3e-g** show the differences in surface structure between the OM $CeZrO_2$ and the blank monolith, as well as the surface morphology of the OM $CeZrO_2$ before and after the removal of the PS template through calcination. In **Supplementary Fig. 3e**, no regular shapes are observed, while in **Supplementary Fig. 3f**, the sol is seen adhering to the surface of the PS spheres. Both are uniformly coated on the cordierite substrate surface. In **Supplementary Fig. 3g**, the PS spheres have been removed after high-temperature calcination, showing the OM structure.

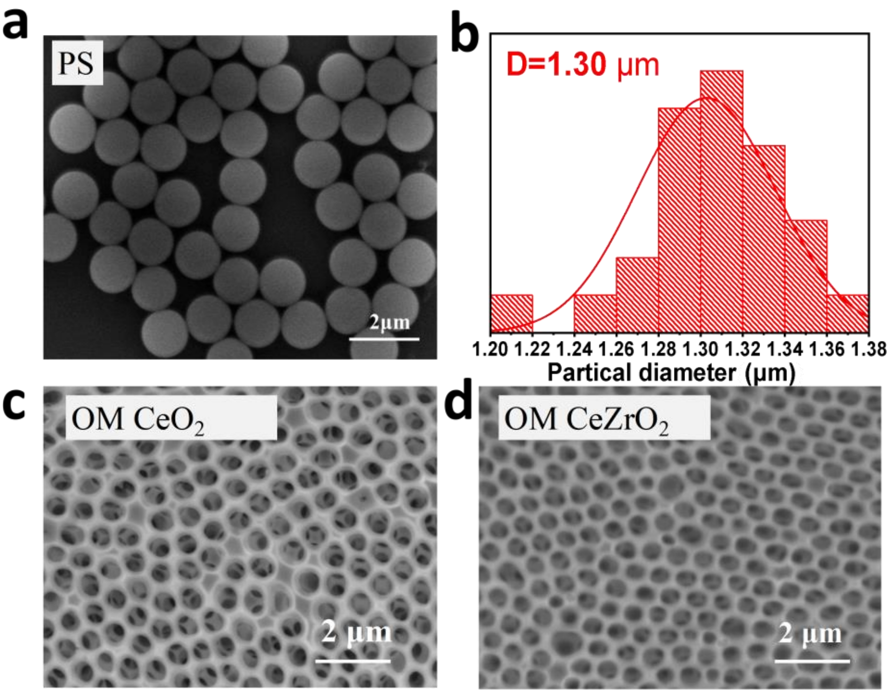

Supplementary Fig. 4: (**a**) SEM images of PS template; (**b**) The particle size distribution of PS template; (**c**, **d**) Top view SEM images of OM CeO_2 and OM $CeZrO_2$.

Supplementary Fig. 4 shows that the particle size of the PS spheres is around 1.3 μm. Both OM CeO_2 and OM $CeZrO_2$ exhibit a well-formed OM structure, with pore sizes around 0.7 μm. The smaller OM pore size compared to the PS particle size is attributed to the shrinkage that occurs during the high-temperature calcination process.

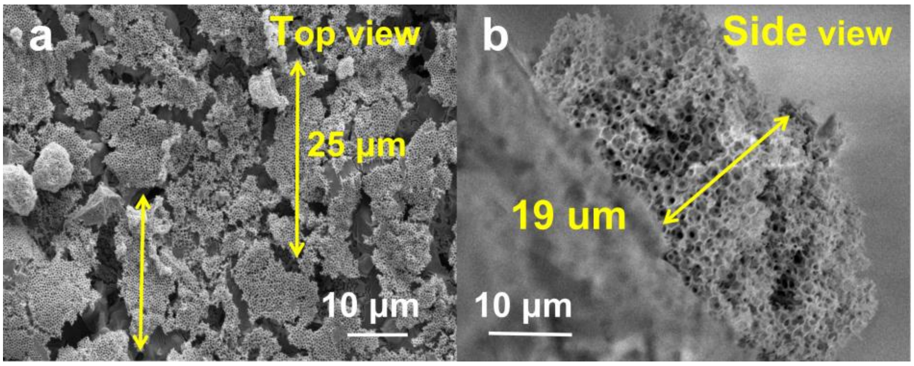

Supplementary Fig. 5: (**a**) Top view and (**b**) cross-sectional SEM images of OM Pt_{SA}/CeO_2.

Supplementary Fig. 5a shows that the OM CeO_2 layer is uniformly applied to the surface of the cordierite substrate, achieving almost complete coverage. The thickness of the OM CeO_2 coating is approximately 19 µm, which is similar to that of OM $CeZrO_2$ (**Supplementary Fig. 5b**).

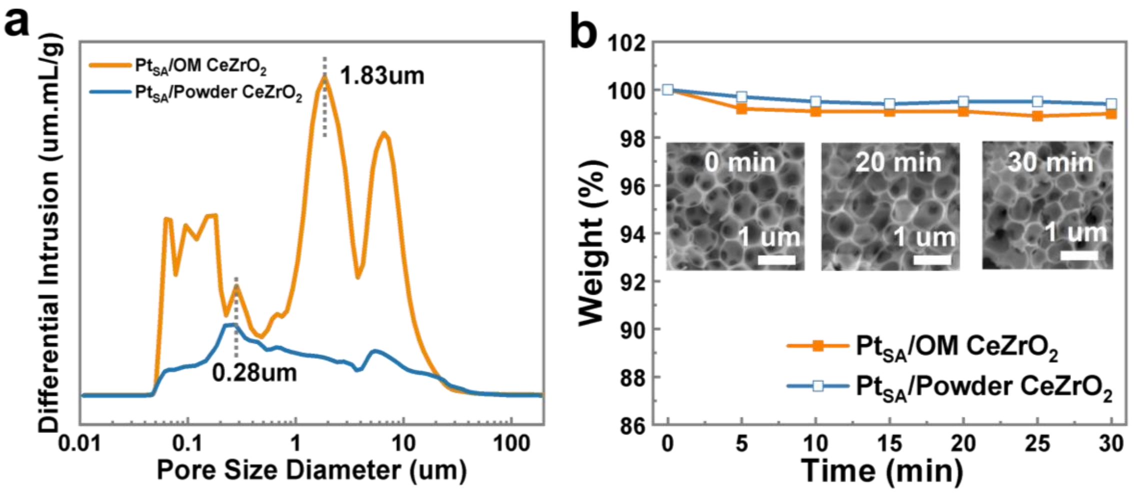

Supplementary Fig. 6: (**a**) Pore size distributions determined by Mercury intrusion porosimetry (MIP); (**b**) Mechanical stability of Pt_{SA}/OM $CeZrO_2$，the inside shows the SEM images of the samples after ultrasound treatment for different durations.

As shown in **Supplementary Fig. 6a**, the mass loss of the OM sample during ultrasound treatment is slightly higher than that of the Powder sample, possibly due to minor damage to the OM pore structure. However, the mass loss difference between the two samples is minimal, with both being less than 1%. This suggests that the OM coating exhibits good mechanical stability, with no significant difference compared to traditional coatings. Furthermore, the SEM images (**Supplementary Fig. 6b** inside) of the Pt_{SA}/OM $CeZrO_2$ catalyst after ultrasound treatment for different durations show that the OM structure undergoes slight deformation, but the overall OM framework structure remains stable, further confirming the excellent mechanical stability of the Pt_{SA}/OM $CeZrO_2$ catalyst. The good mechanical stability of the Pt_{SA}/OM $CeZrO_2$ catalyst may be attributed to its uniform and ordered pore structure, which allows stress to be evenly distributed across the surface and within the pores, rather than concentrating in a specific area, thereby better maintaining overall stability and reducing local collapse.

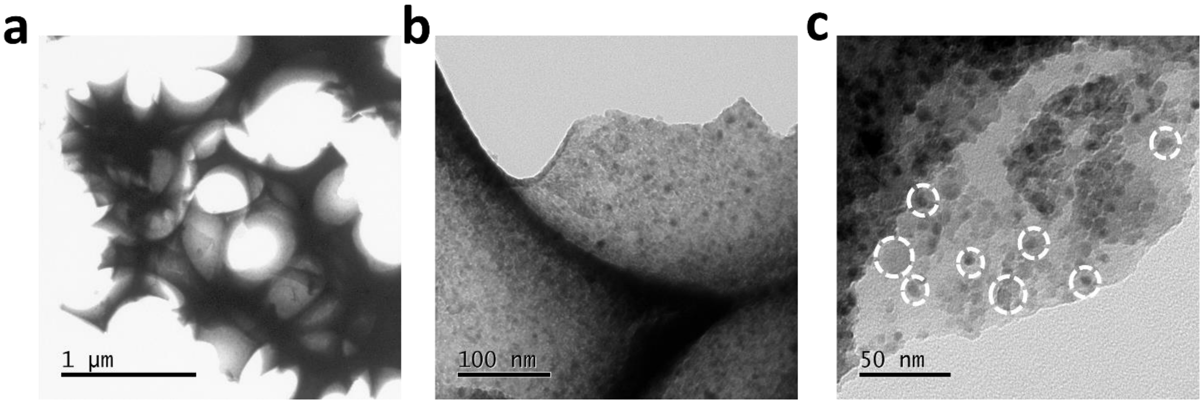

Supplementary Fig. 7: TEM images of OM $CeZrO_2$ at different magnifications.

As shown in **Supplementary Fig. 7**, OM $CeZrO_2$ is formed by the aggregation of Ce-Zr solid solution nanoparticles with particle sizes ranging from 5 to 30 nm.

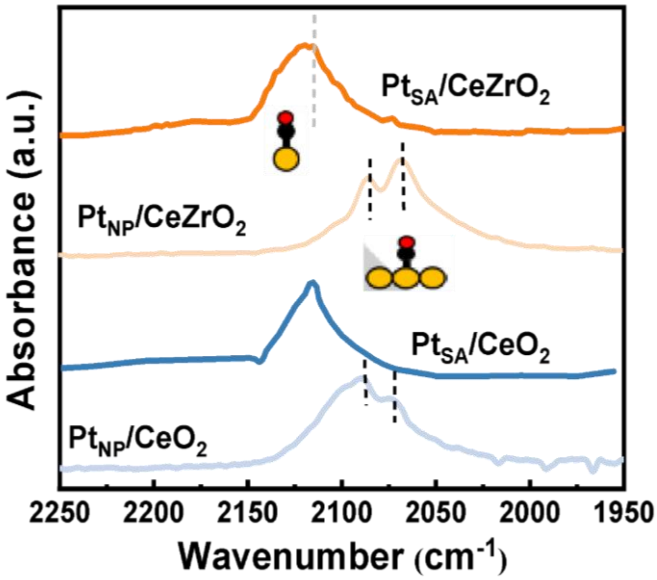

Supplementary Fig. 8: CO-DRIFTS of catalysts.

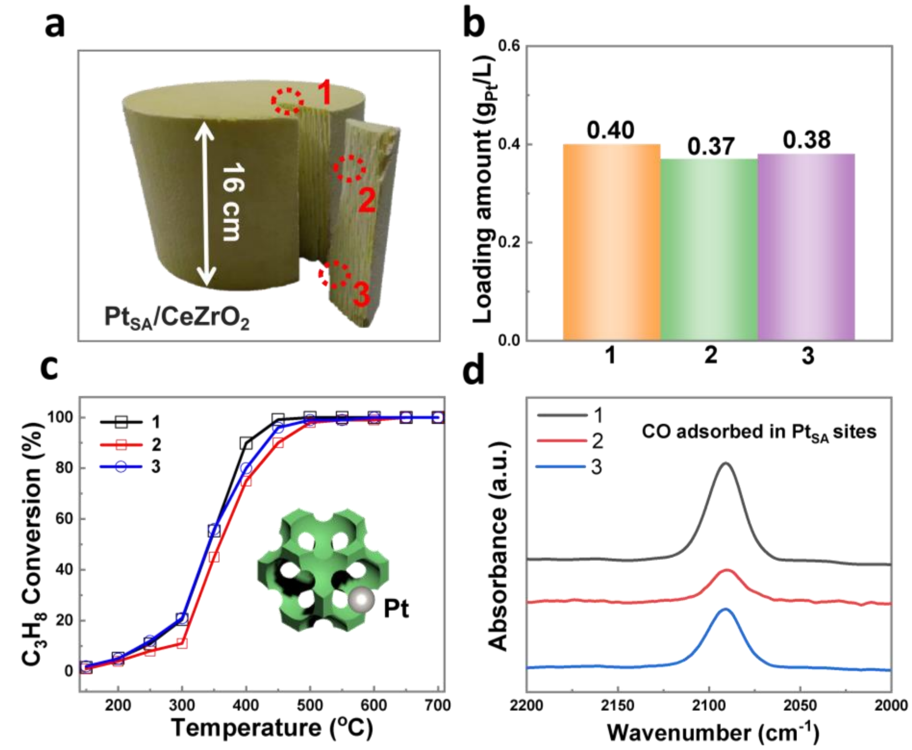

Supplementary Fig. 9: (a) large-scale $Pt_{SA}/CeZrO_2$ catalytic device; (b) Pt loading amount form ICP data; (c) catalytic activity and (d) CO-DRIFTS across these positions.

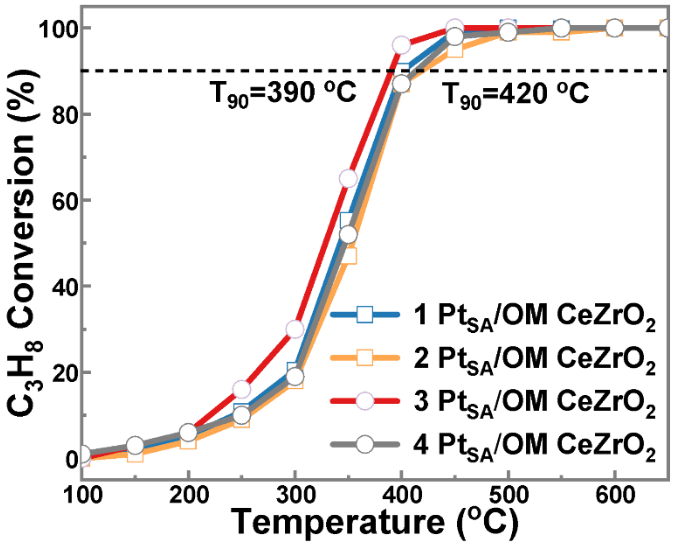

Supplementary Fig. 10 Performance testing data for multiple catalysts from different batches.

To investigate the reproducibility of catalyst preparation, we evaluated the propane catalytic oxidation performance across four catalyst batches, showing T_{90} values around 405 °C. While achieving complete consistency in performance across different batches remains challenging due to difficulties in precisely controlling the uniformity of the monolithic catalyst's ordered macroporous (OM) pore structure and Pt loading, rigorous optimization of synthesis protocols enabled us to constrain the T_{90} error margin within ±15 °C. This result demonstrates satisfactory reproducibility of the catalyst.

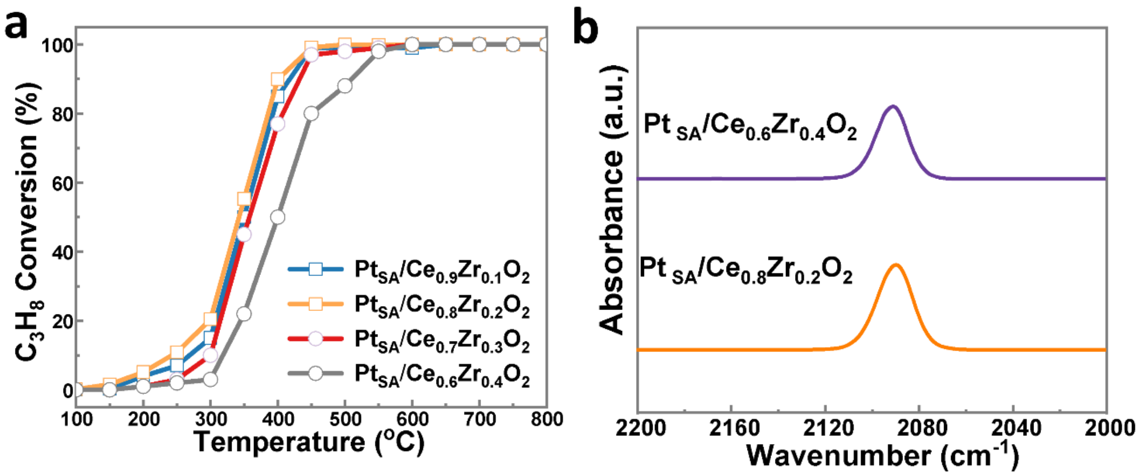

Supplementary Fig. 11: (**a**) Light-off curves for $Pt_{SA}/Ce_{0.9}Zr_{0.1}O_2$, $Pt_{SA}/Ce_{0.8}Zr_{0.2}O_2$, $Pt_{SA}/Ce_{0.7}Zr_{0.3}O_2$ and $Pt_{SA}/Ce_{0.6}Zr_{0.4}O_2$; (b) CO DRIFTS for $Pt_{SA}/Ce_{0.8}Zr_{0.2}O_2$ and $Pt_{SA}/Ce_{0.6}Zr_{0.4}O_2$.

We also prepared catalysts with different Zr doping ratios: $Pt_{SA}/Ce_{0.9}Zr_{0.1}O_2$, $Pt_{SA}/Ce_{0.8}Zr_{0.2}O_2$, $Pt_{SA}/Ce_{0.7}Zr_{0.3}O_2$, $Pt_{SA}/Ce_{0.6}Zr_{0.4}O_2$. The activity test results (**Supplementary Fig. 11a**) show that $Pt_{SA}/Ce_{0.8}Zr_{0.2}O_2$ exhibits the best C_3H_8 catalytic oxidation activity (T_{90} =400 °C), while $Pt_{SA}/Ce_{0.6}Zr_{0.4}O_2$ shows the poorest catalytic oxidation activity (T_{90} = 530 °C). It is evident that as the Zr doping ratio increases to over 30 %, the catalytic activity decreases significantly. The superior performance of $Pt_{SA}/Ce_{0.8}Zr_{0.2}O_2$ is likely due to the fact that it ensures Pt_{SA} is primarily loaded around Zr. In contrast, a low Zr content (< 0.1) may not be sufficient to affect the coordination structure of most Pt_{SA}. Additionally, excessive Zr doping (> 0.3) reduces the surface Ce^{3+} species with variable valence, thereby inhibiting the catalytic oxidation reaction.[1] Therefore, $Pt_{SA}/Ce_{0.8}Zr_{0.2}O_2$ was selected for subsequent research. To study the effect of the Zr doping ratio on the Pt_{SA} structure, we characterized $Pt_{SA}/Ce_{0.8}Zr_{0.2}O_2$ and $Pt_{SA}/Ce_{0.6}Zr_{0.4}O_2$ using CO-DRIFTS, as shown in the **Supplementary Fig. 11b**. Both samples exhibit CO adsorption peaks only in the range of 2101-2112 cm^{-1}, corresponding to the characteristic peak of CO adsorbed at Pt single-atom sites. This indicates that even when the Zr doping ratio is increased to 0.4, the single-atom structure of Pt can still be maintained.

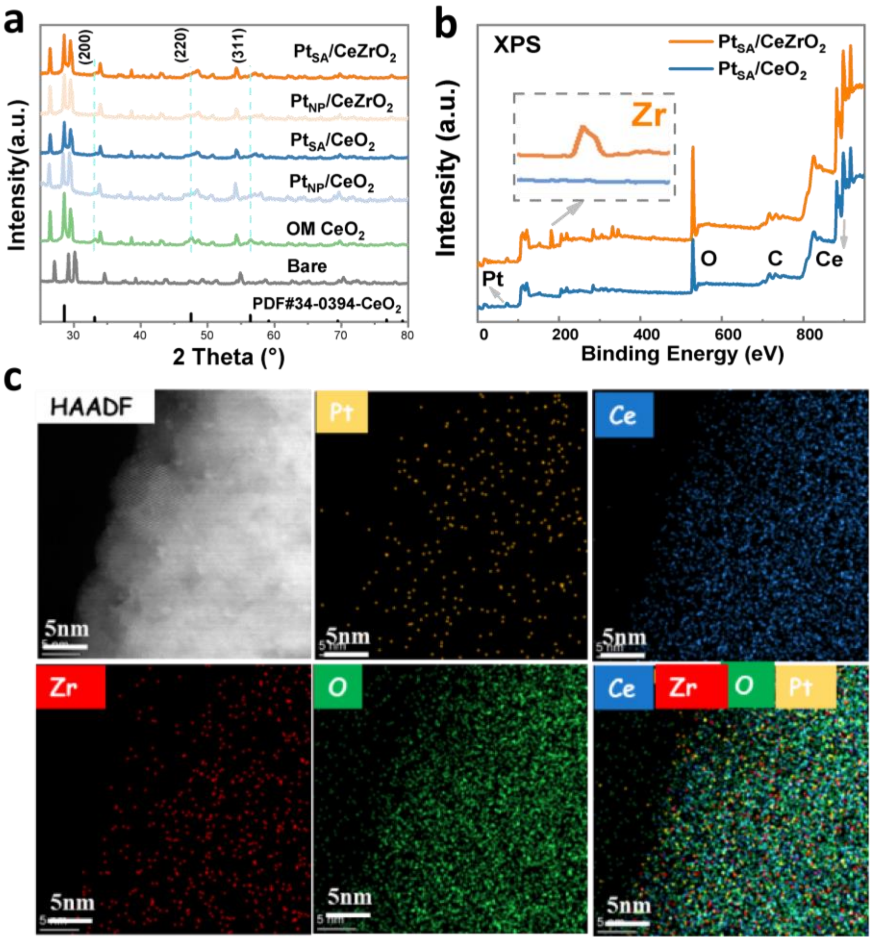

Supplementary Fig. 12: (**a**) XRD patterns of catalysts; (**b**) XPS full spectra of catalyst; (**c**) EDX-Mapping of $Pt_{NP}/CeZrO_2$.

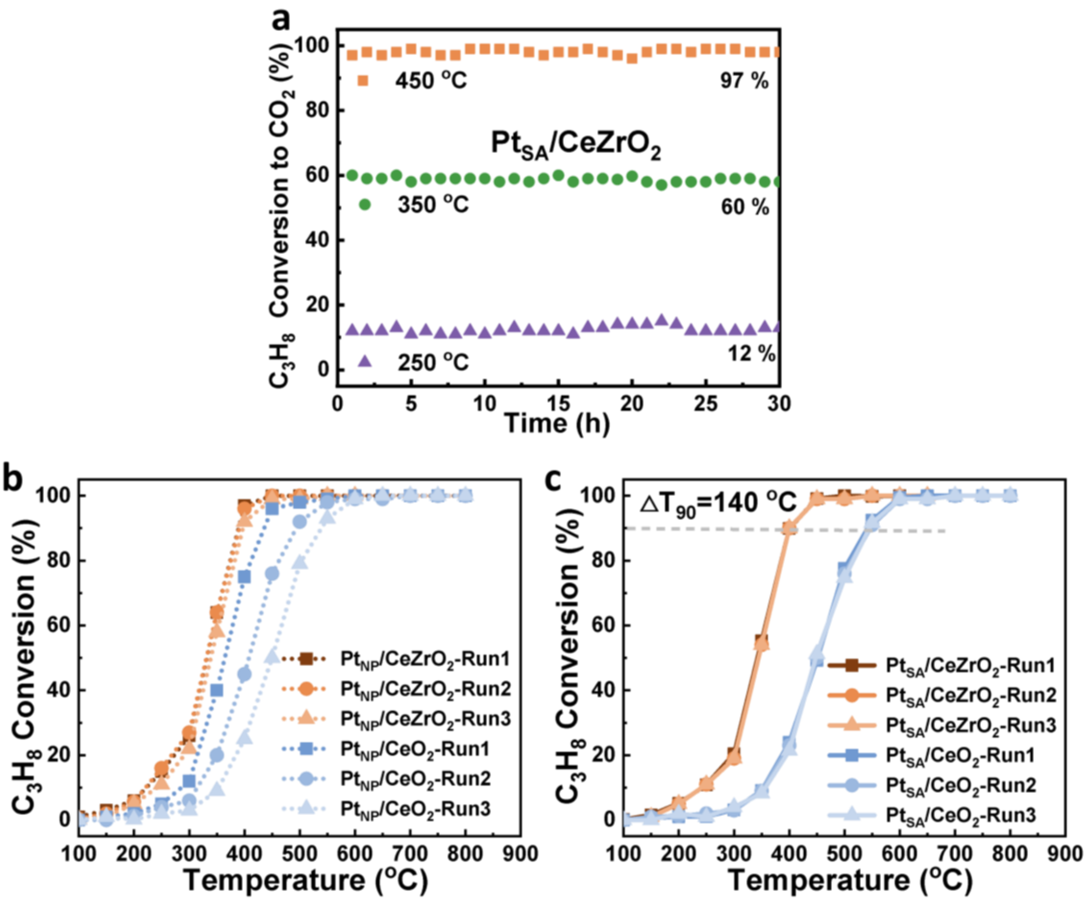

Supplementary Fig. 13: (**a**) Stability performance of catalysts under different temperature; (**b, c**) Cycling stability performance of catalysts.

As shown in **Supplementary Fig. 13b**, $Pt_{NP}/CeZrO_2$ exhibits good cyclic stability, while the activity of Pt_{NP}/CeO_2 significantly decreases during the cycling tests. This could be attributed to the highest testing temperature of 800 °C, at which both catalysts may transform into Pt_{SA}. As shown in **Fig. 2a**, after thermal aging at 800 °C, Pt_{SA}/CeO_2 shows poor activity, whereas $Pt_{SA}/CeZrO_2$ maintains activity similar to that of $Pt_{NP}/CeZrO_2$. These results further confirm the transformation of Pt_{NP} to Pt_{SA} at 800 °C and highlight the poor high-temperature resistance of Pt_{NP}/CeO_2. As seen in **Supplementary Fig. 13c**, both Pt_{SA} catalysts obtained after 800 °C thermal aging exhibit good cyclic stability, but the activity of Pt_{SA}/CeO_2 is significantly lower than that of $Pt_{SA}/CeZrO_2$, with a T_{50} increase of approximately 100 °C, further demonstrating its poor resistance to 800 °C.

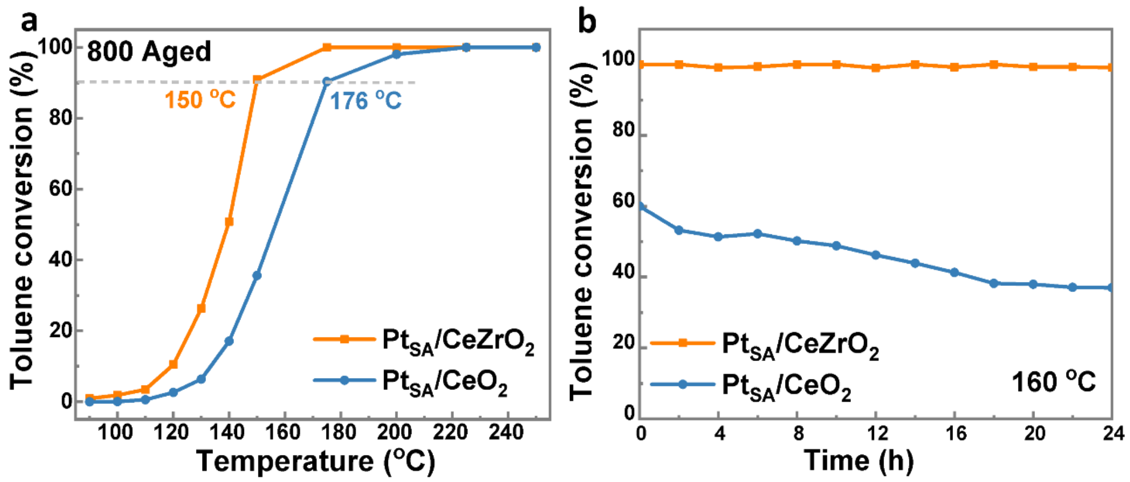

Supplementary Fig. 14: (**a**) Light-off curves for aged Pt_{SA}/CeO_2, $Pt_{SA}/CeZrO_2$ (Aging conditions: 800 °C for 50 hours in air). (**b**) Durability performance at 160 °C.

As shown in **Supplementary Fig. 14a**, the activity test demonstrates that the T_{90} for toluene oxidation over $Pt_{SA}/CeZrO_2$ after aging is approximately 25 °C lower than that of Pt_{SA}/CeO_2 (176 °C → 150 °C). Additionally, in the stability test at 160 °C (**Supplementary Fig. 14b**), $Pt_{SA}/CeZrO_2$ also exhibited good activity and stability, achieving 100 % operation with no decay over 24 hours.

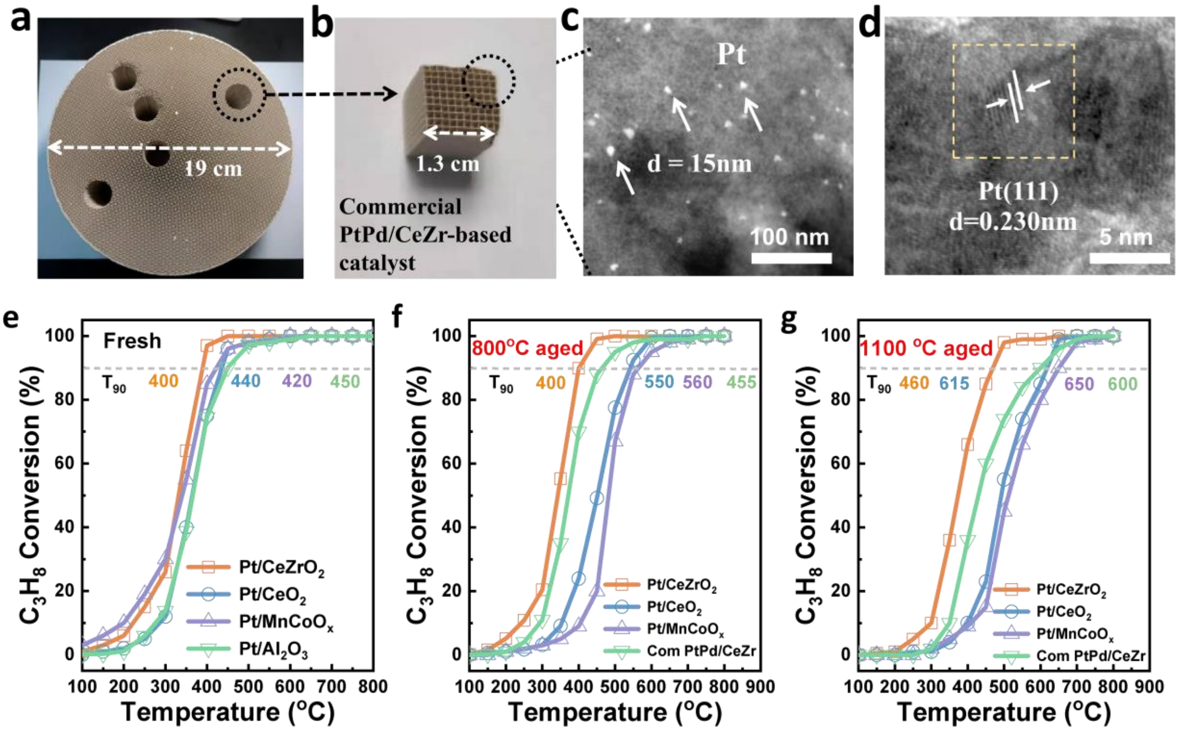

Supplementary Fig. 15: (**a, b**) Actual photographs of the commercial PtPd/CeZr based catalyst; (**c, d**) TEM images of Com-PtPd/CeZr; (**e-g**) Stability comparison of $Pt_{SA}/CeZrO_2$ with commercial $Pt/MnCoO_x$ and Com-PtPd/CeZr catalysts after 800 °C aged for 50 h or 1000 °C for 2h in flowing air.

As shown in **Supplementary Fig. 15a** and **b**, small sections cut from commercial PtPd/CeZr-based catalysts are used for subsequent comparisons of activity and stability. The TEM results indicate that the particle size of Pt is around 15 nm (**Supplementary Fig. 15c** and **d**).

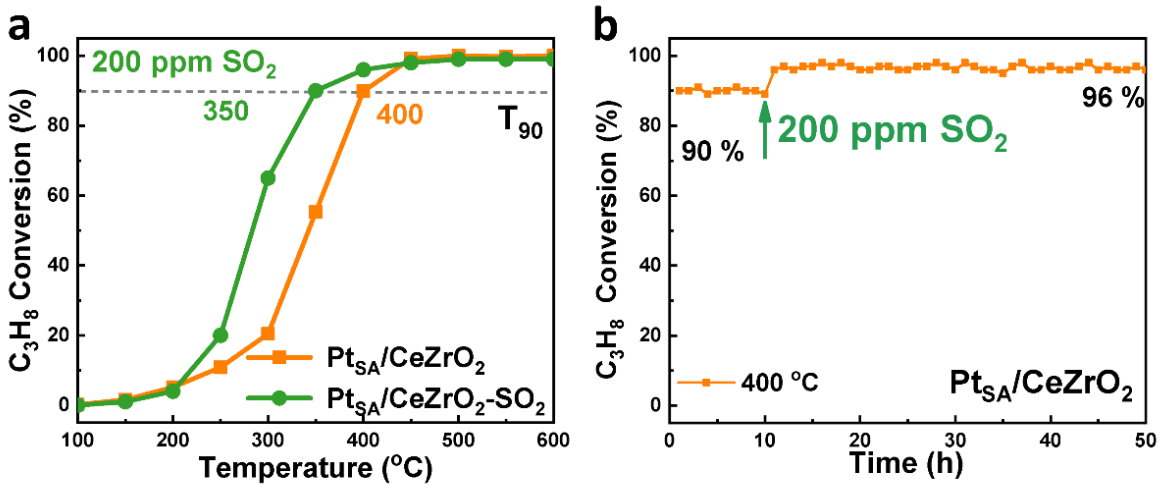

Supplementary Fig. 16 (a) C_3H_8 catalytic oxidation activity of $Pt_{SA}/CeZrO_2$ catalyst under different reaction conditions (with or without SO_2); (b) C_3H_8 catalytic oxidation sulfur resistance lifetime test of $Pt_{SA}/CeZrO_2$ catalyst at 400 °C with 200 ppm SO_2 in the reaction atmosphere.

Supplementary Fig. 16 shows the sulfur resistance performance of the $Pt_{SA}/CeZrO_2$ catalyst in C_3H_8 catalytic oxidation. Notably, after introducing 200 ppm SO_2, the catalytic oxidation conversion increased, with the T_{90} decreasing from 400 °C to 350 °C (**Supplementary Fig. 16a**). Additionally, at 400 °C, the conversion improved from 90% to 96% upon the introduction of 200 ppm SO_2, maintaining stable performance for 50 hours without any degradation (**Supplementary Fig. 16b**). The introduction of SO_2 would induce a strong coordination interaction between Pt species and SO_4^{2-}, and the two can work synergistically to break the C-C and C-H bonds in C_3H_8.[2] These results highlight the excellent sulfur resistance of the $Pt_{SA}/CeZrO_2$ catalyst.

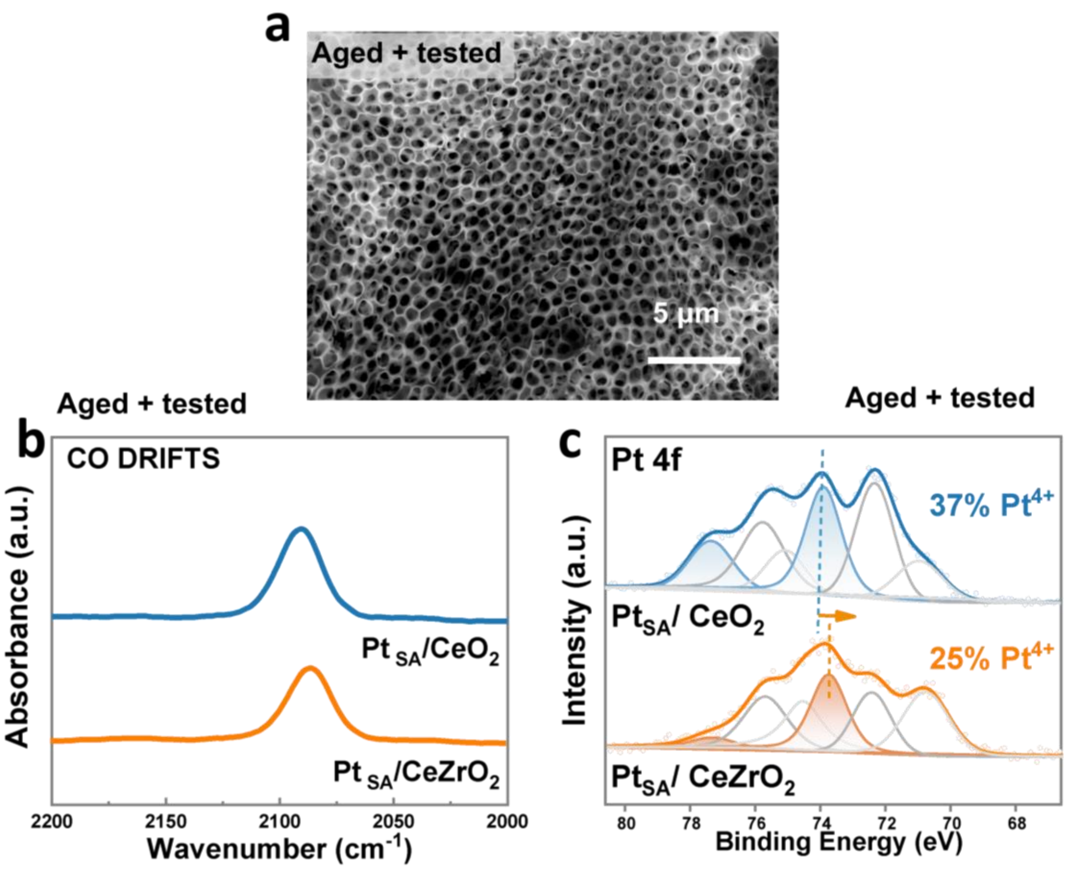

Supplementary Fig. 17: (**a**) SEM images of aged + tested Pt_{SA}/CeO_2 after aging and testing in Figure 2d; (**b**) CO-DRIFTS and (**c**) XPS Pt4f results of aged + tested samples.

Both aged + tested Pt_{SA}/CeO_2 and $Pt_{SA}/CeZrO_2$ had only a linear CO peak at 2101-2112 cm^{-1} in CO-DRIFTS results (**Supplementary Fig. 17b)**, indicating that a CO molecule was adsorbed on isolated Pt single atom site.

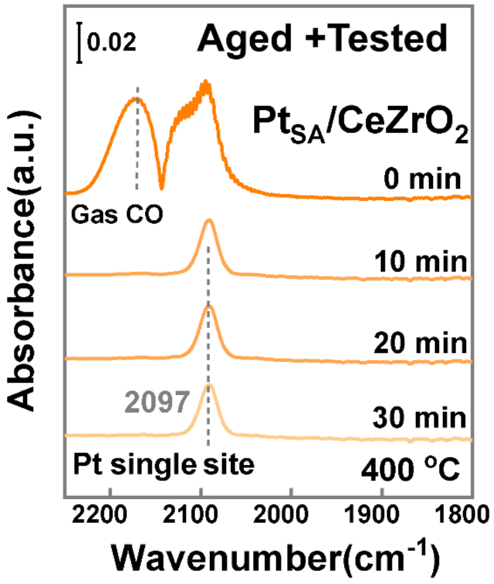

Supplementary Fig. 18 The *in situ* CO DRIFTS of $Pt_{SA}/CeZrO_2$ after 400 °C, $1C_3H_8$, 20 O_2).

We conducted in situ CO-DRIFTS experiments on the aged and tested $Pt_{SA}/CeZrO_2$ catalyst at the T_{90} reaction temperature (400 °C). As shown in **Supplementary Fig. 18**, after switching off the CO gas supply, only a peak appears at 2097 cm^{-1}, indicating that CO is chemically adsorbed on the Pt single-atom sites and remains stable at 400 °C, without showing any peak related to CO bridging adsorption on Pt nanoparticles. The *in situ* CO-DRIFTS further corroborated the conclusions drawn from STEM and *ex situ* CO-DRIFTS (**Fig. 2g and Supplementary Fig. 17b**), showing that both $Pt_{SA}/CeZrO_2$ and Pt_{SA}/CeO_2 catalysts have excellent sintering resistance.

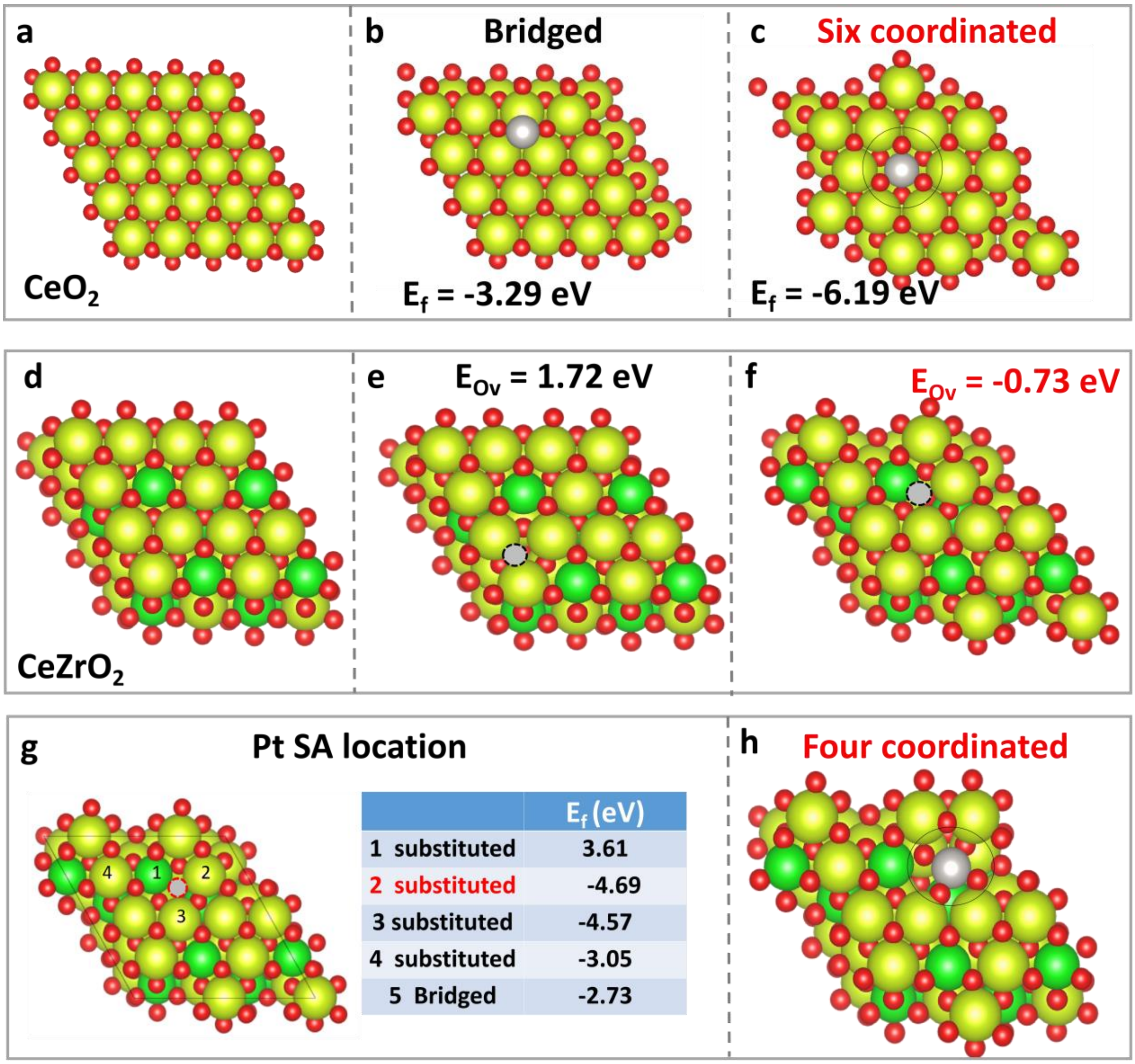

Supplementary Fig. 19: (**a**) CeO_2 model; (**b, c**) The Pt_{SA}/CeO_2 model; (**d-f**) $CeZrO_2$ model; (**g**) the Pt_{SA} loading position optimize; (**h**) The $Pt_{SA}/CeZrO_2$ model.

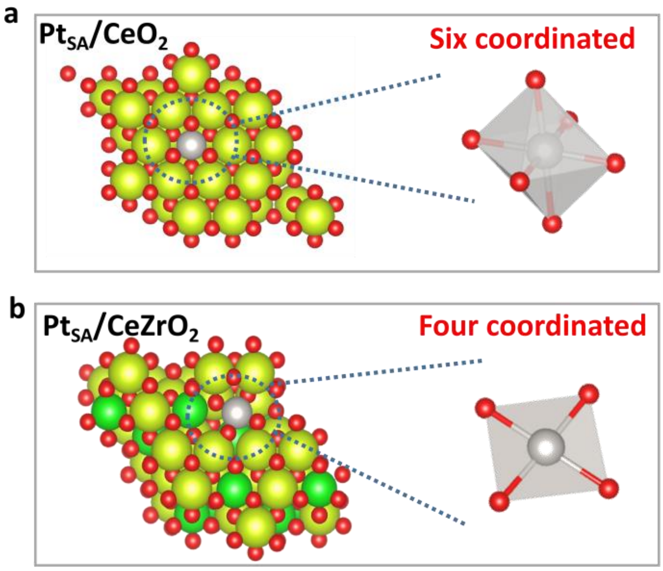

Supplementary Fig. 20: The most thermodynamically stable configurations of Pt_{SA}/CeO_2 and $Pt_{SA}/CeZrO_2$.

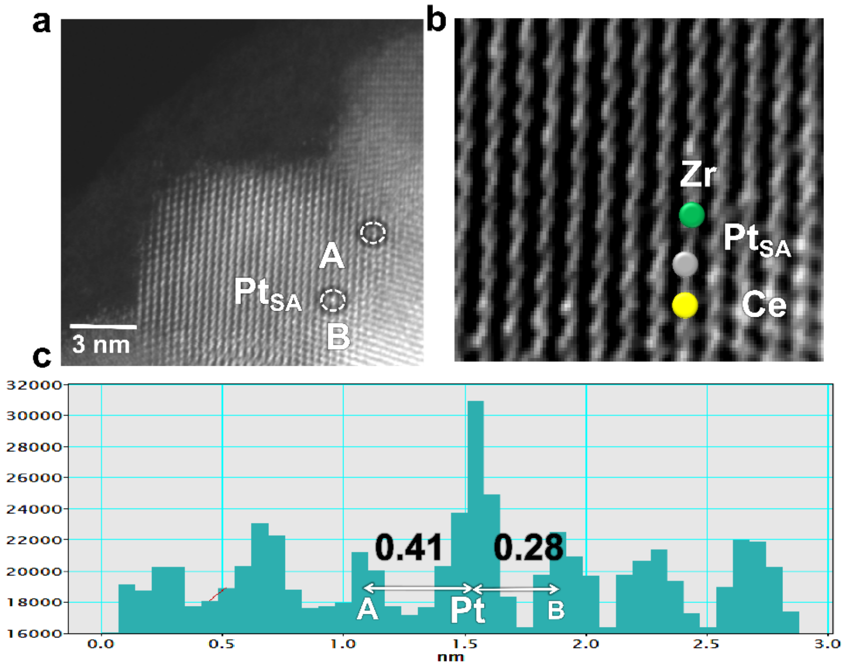

Supplementary Fig. 21: Annular Dark Field Scanning Transmission Electron Microscopy (ac-HADDF STEM) images of aged atomically dispersed $Pt_{SA}/CeZrO_2$.

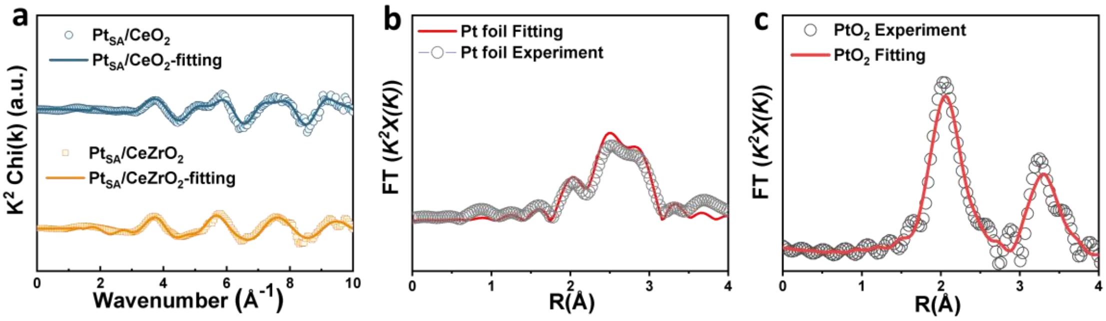

Supplementary Fig. 22: EXAFS fitting curves in (**a**) k-space at the Pt-L3 edge and (**b, c**) R-space. The detailed fitting results can be found in **Supplementary Table 1**.

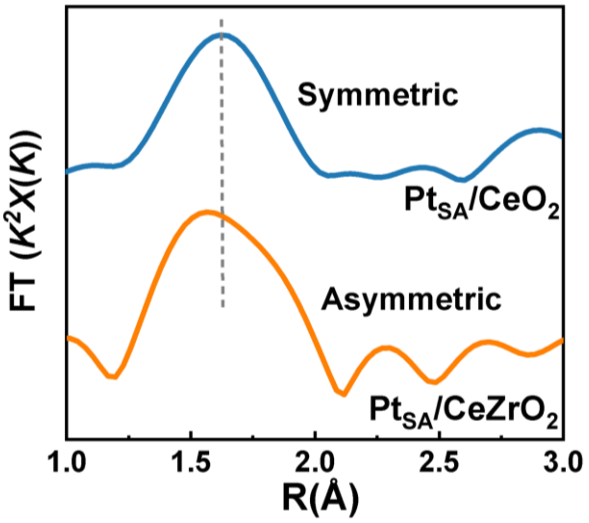

Supplementary Fig. 23: EXAFS data for aged Pt_{SA}/CeO_2 and $Pt_{SA}/CeZrO_2$.

As shown in **Fig. 3c-d**, **Supplementary Table 3**, and **Supplementary Fig. 23**, the first shell of Pt_{SA} on CeO_2 consists of six Pt-$O_{(Ce)}$ bonds of the same length, producing a symmetric Pt-O peak. In contrast, the first shell of Pt_{SA} on $Ce_{0.8}Zr_{0.2}O_2$ has three shorter Pt-$O_{(Ce)}$ bonds at 1.67 Å and one longer Pt-$O_{(Zr)}$ bond at 1.97 Å. The distortion arises from the different ionic radii of Ce^{4+} (~0.92 Å) and Zr^{4+} (~0.80 Å), which induce local strain and alter Pt–O bond lengths. These similar but differing bond lengths result in an asymmetric Pt-O peak, further supporting that Pt is loaded near Zr atoms on $Pt_{SA}/CeZrO_2$.

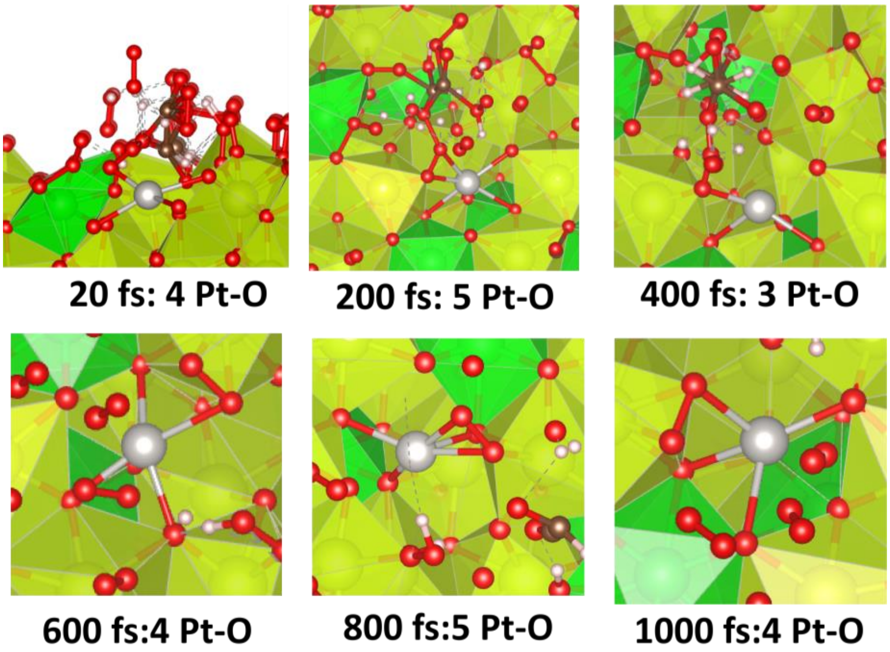

Supplementary Fig. 24: The snapshots of $Pt_{SA}/CeZrO_2$ model from the AIMD simulation at different time (400 ºC, $1C_3H_8$, 20 O_2).

As shown in **Supplementary Fig. 24**, the coordination number of the Pt-O bond fluctuates between 4 and 5 throughout most of the reaction under 400 ºC.

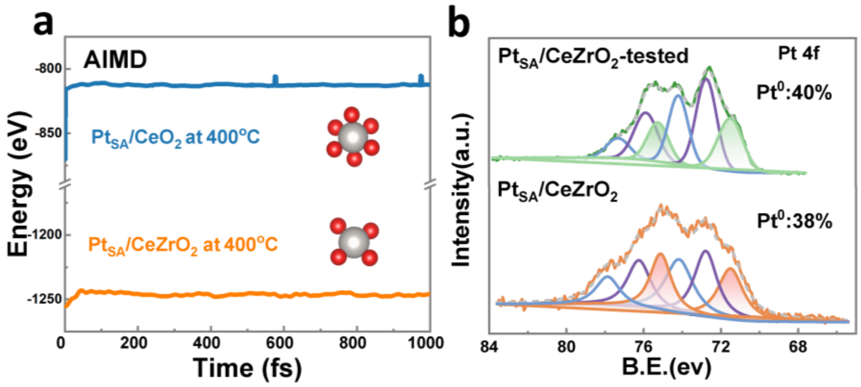

Supplementary Fig. 25: (**a**) Ab-initio molecular dynamics (AIMD) simulations under reaction condition (1 C_3H_8: 20 O_2; at 400 °C); (**b**) Pt 4f XPS spectra of fresh and tested $Pt_{SA}/CeZrO_2$.

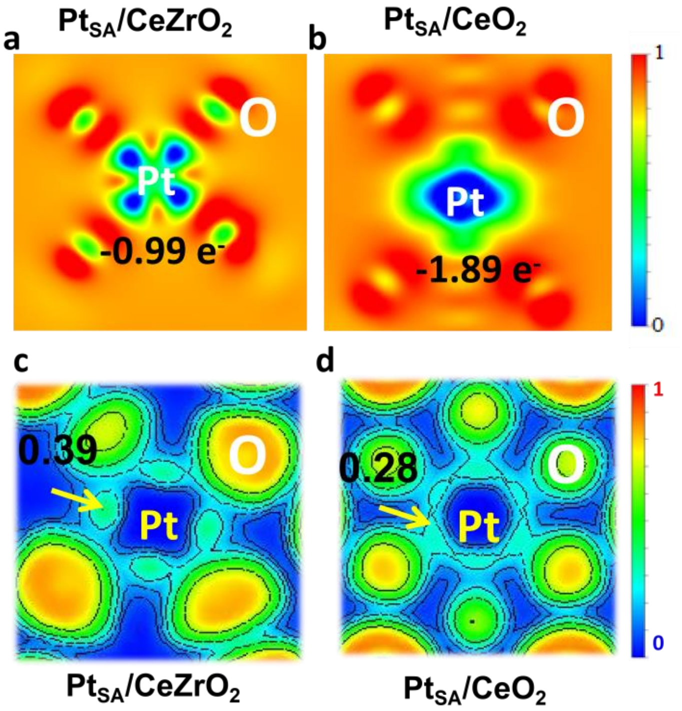

Supplementary Fig. 26: Calculated charge differential density of (**a**) Pt$_{SA}$/CeZrO$_2$ and (**b**) Pt$_{SA}$/CeO$_2$; The electronic local function (ELF) spectra of (**c**) Pt$_{SA}$/CeZrO$_2$ and (**d**) Pt$_{SA}$/CeO$_2$.

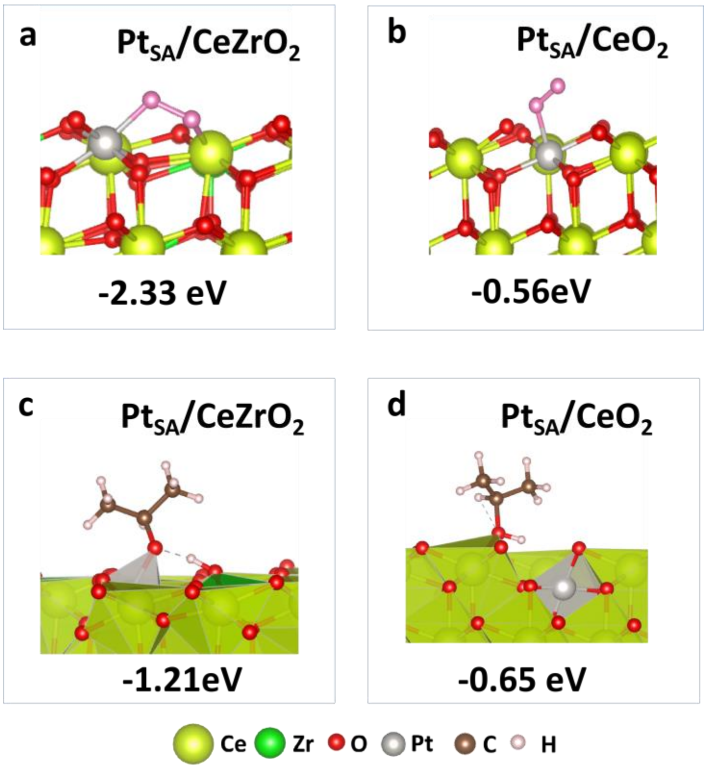

Supplementary Fig. 27: The O_2 (**a, b**) and C_3H_8 (**c, d**) adsorption energy on $Pt_{SA}/CeZrO_2$ and Pt_{SA}/CeO_2.

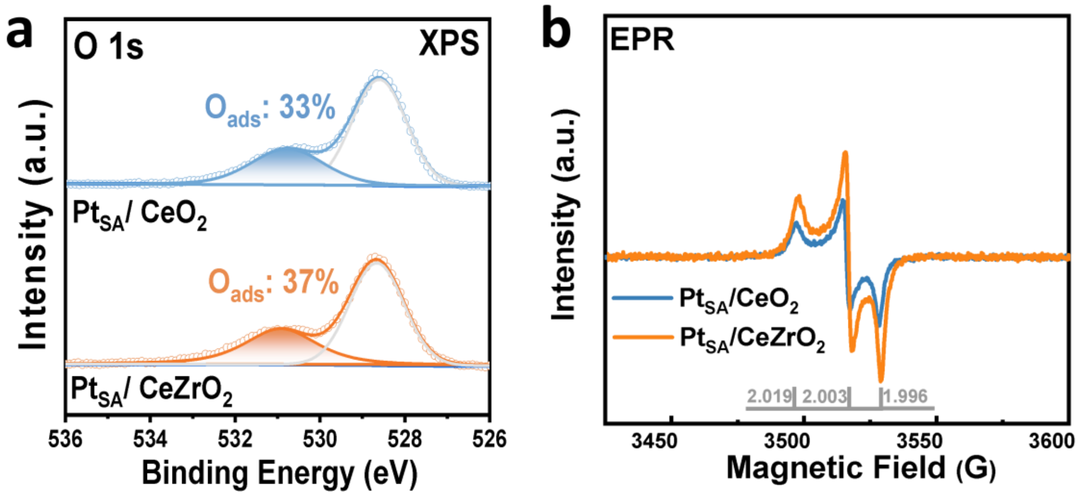

Supplementary Fig. 28: The O 1s XPS spectra (**a**) and low-temperature EPR spectra (**b**) in air of $Pt_{SA}/CeZrO_2$ and Pt_{SA}/CeO_2.

As shown in EPR experiments in air (**Supplementary Fig. 28b**), both Pt_{SA}/CeO_2 and $Pt_{SA}/CeZrO_2$ catalysts exhibited a hyperfine structure with three parameters at $g_1 = 2.019$, $g_2 = 2.003$, and $g_3 = 1.996$, respectively. These EPR peaks can be assigned to adsorbed O^- radicals. The EPR detective O^- radical is the principal fragment of the chemisorbed peroxide O_2^{2-} species because the chemisorbed peroxide O_2^{2-} species tends to partially dissociate to O^- species (EPR silent). Accordingly, the emergence of the O^- EPR signal suggests the existence of O_2^{2-} species.[3,4] The intensity of the EPR signal on the $Pt_{SA}/CeZrO_2$ catalyst is stronger than that of the Pt_{SA}/CeO_2 catalyst, further indicating higher concentration of the chemisorbed peroxide O_2^{2-} species on the surface of $Pt_{SA}/CeZrO_2$.

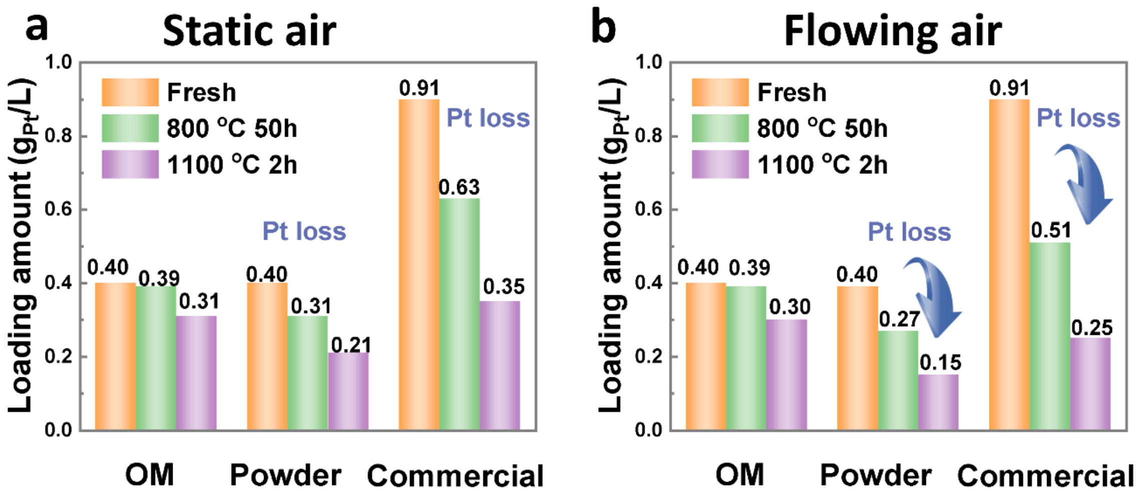

Supplementary Fig. 29: (a, b) Changes in Pt loading of OM Pt/$CeZrO_2$, Powder Pt/$CeZrO_2$, Com-PtPd/CeZr catalyst after aging at 800 °C and 1100 °C in static air (a) and flowing air (b). (20% O_2, N_2 balance, flow rate 50 mL/min, 800 °C for 50 hours / 1000 °C for 2 hours).

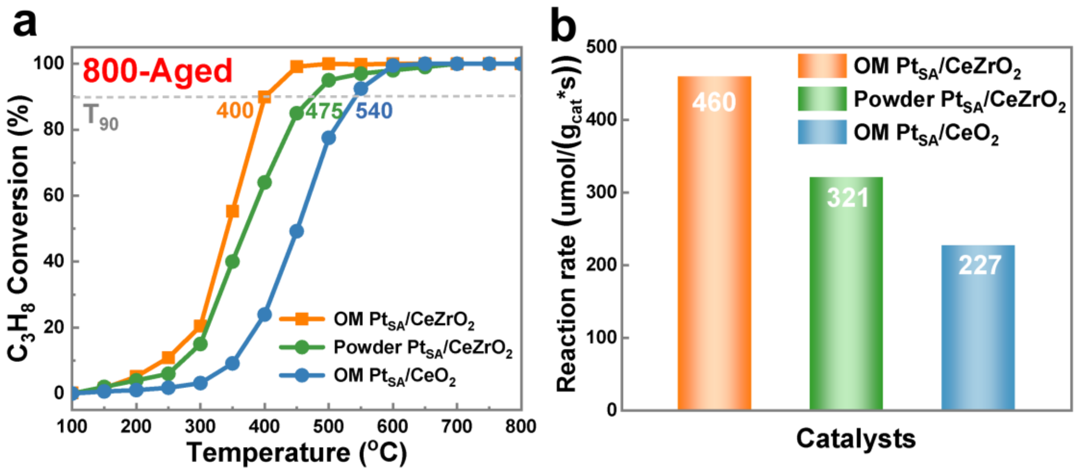

Supplementary Fig. 30: Catalytic performance of aged OM $Pt_{SA}/CeZrO_2$, OM Pt_{SA}/CeO_2 and Powder $Pt_{SA}/CeZrO_2$. (**a**) Light-off curves and (**b**) Normalized reaction rate at 450 ºC. (Aging conditions: 800 °C for 50 hours in air).

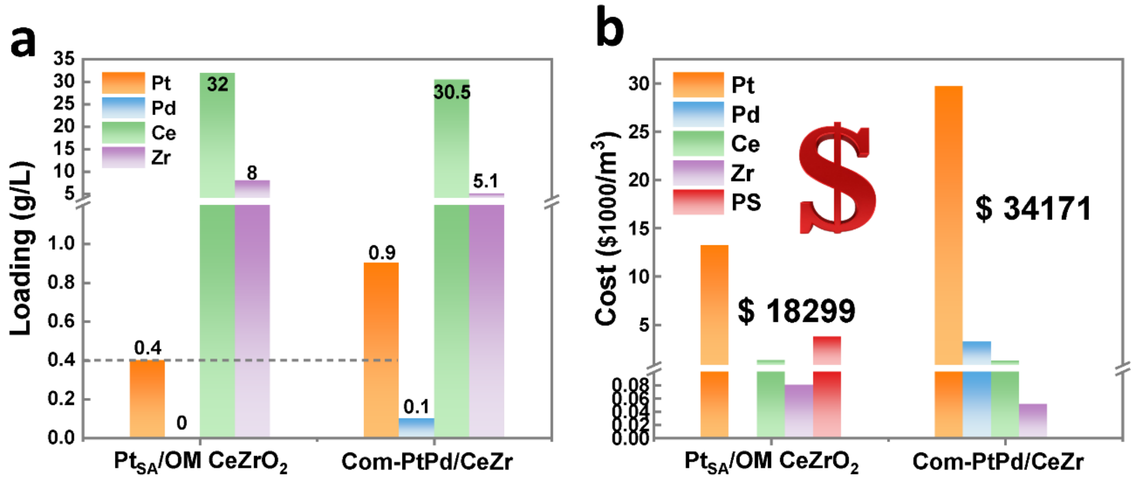

Supplementary Fig. 31: (**a**) Loading amount and (**b**) Material consumption costs comparison between the Pt_{SA}/OM $CeZrO_2$ integrated monolith (0.4 g_{Pt}/L) and commercial PtPd/CeZr based catalysts (0.9 g_{Pt}/L+ 0.1g_{Pd}/L).

First, we compared the raw material consumption cost per cubic meter for the Pt_{SA}/OM $CeZrO_2$ integrated monolith and commercial PtPd/CeZr-based catalysts based on their catalyst loadings (**Supplementary Fig. 31a**) and metal prices as of March 29, 2025 (Pt: \$33/g, Pd: \$33/g, Ce: \$0.04/g, Zr: \$0.01/g), including the consumption cost of PS microsphere materials (styrene, polyvinylpyrrolidone, and azo-bis-isobutyronitrile) at \$3.73/L. As shown in **Supplementary Fig. 31b**, the raw material consumption cost of Pt_{SA}/OM $CeZrO_2$ was reduced by 46%, from \$34,171/m^3 to \$18,299/m^3.

This technology can use conventional coating process equipment, so the equipment cost is similar to that of traditional catalysts. However, the drying and calcination steps may be more complex than traditional coating methods (to ensure uniformity in the OM structure and Pt loading), requiring more precise control, which could lead to a slight increase in processing costs. The production cost of PS microsphere raw materials is relatively low and has industrial production potential, but since large-scale production has not yet been realized, it may lead to higher production costs. In the future, it will be necessary to develop large-scale PS microsphere

production technology or explore more economical alternatives such as activated carbon microspheres.

The energy consumption during the drying and calcination steps in this technology does not differ significantly from that of traditional washcoat methods. Microwave drying, though more power-intensive, only lasts for half a minute. The PS template removal process is integrated into the crystallization process of $CeZrO_2$ and only requires a slightly extended time during the low-temperature phase. However, the cost savings from the reduced amount of precious metal Pt should offset these additional consumptions, making the catalyst more economical.

The catalyst reuse and recovery methods are the same as those of traditional catalysts (mainly high-temperature reduction and acid leaching), and due to the excellent stability performance of this catalyst, significant savings in catalyst regeneration and replacement costs can be achieved.

Supplementary Tables

Supplementary Table 1. Comparative activity of catalysts for the catalytic oxidation of C_3H_8.

Catalysts	concentration (ppm)	Space velocity ($mL \cdot g^{-1} \cdot h^{-1}$)	T_{50}/T_{90} (°C)	Ref
$Pt_{SA}/CeZrO_2$	3000	37500	345/400	This work (800 ºC-Aged)
Pt_{SA}/CeO_2	3000	37500	450/550	This work (800 ºC-Aged)
PtPd/CeZr	3000	37500	370/455	Commercial (800 ºC-Aged)
$Pt/MnCoO_x$	3000	37500	490/560	Commercial (800 ºC-Aged)
Pt/TiO_{2-x}	10000	60000	550/650	3
Pt/CeO_2	1000	12000	375/465	5
Pd/CeO_2	2000	30000	325/530	6
$Pt/LaCoO_3$	8000	30000	390/500	7
Pt/CeO_2-HA	2000	36000	320/450	8

Supplementary Table 1 shows that $Pt_{SA}/CeZrO_2$ exhibits the lowest T_{90} for C_3H_8 oxidation compared with other Pt-based catalysts reported in the references, even after thermal aging at 800 °C.

Supplementary Table 2. Comparative TOF of catalysts for the catalytic oxidation of C_3H_8.

Catalysts	Pt loading (wt %)[a]	Reaction rate (μmol/(g_{cat}*s))[b]	TOF*10^3 (s^{-1})[b]	Ref
$Pt_{SA}/CeZrO_2$	0.04	102.7	501	This work (800 ºC-Aged)
Pt_{SA}/CeO_2	0.04	4.5	22	This work (800 ºC-Aged)
$Pt/MnCoO_x$	0.04	3.5	17	Commercial (800 ºC-Aged)
Pt/TiO_{2-x}	0.52	182.1	68	3
Pt/CeO_2	0.23	112.8	96	5
$Pt/LaCoO_3$	0.29	4.5	3	7
Pt/CeO_2-HA	0.84	258.9	60	8

[a] Measured by ICP.

[b] Calculated by the reaction rate at 300 °C.

Supplementary Table 2 shows that $Pt_{SA}/CeZrO_2$ exhibits the largest TOF for C_3H_8 oxidation compared with other Pt-based catalysts reported in the references, even after thermal aging at 800 °C.

Supplementary Table 3. Structural parameters of $Pt_{SA}/CeZrO_2$ and reference samples extracted from the Pt L3-edge EXAFS fitting. (S_0^2=0.8)[a].

Sample	Path	CN	R（Å）	σ^2（10^{-3} Å^2）	R-factor
Pt-foil	Pt-Pt	12	2.76	4.4±1.2	0.003
PtO_2	Pt-O	6	2.01	3.2±0.9	0.011
Pt_{SA}/CeO_2	Pt-O	5.8	1.71	2.32±4.33	0.020
$Pt_{SA}/CeZrO_2$	Pt-O_{Ce}	3.4	1.67	7.02±6.58	0.015
	Pt-O_{Zr}	0.7	1.97	14.15±3.72	0.015

a) S_0^2 was fixed as 0.8 during EXAFS fitting, based on the known structure of Pt foil.

b) CN is the coordination number.

Supplementary References

[1] Yang, X. et al. Large-pore mesoporous CeO_2–ZrO_2 solid solutions with in-pore confined Pt nanoparticles for enhanced CO oxidation. *Small* **15**, 1903058 (2019).

[2] Zhang, B. et al. Diverse effects of SO_2-Induced Pt–O–SO_3 on the catalytic oxidation of C_3H_6 and C_3H_8. *Environ. Sci. Technol.* **58**, 18020–18032 (2024).

[3] Fang, Y. et al. Oxygen vacancy-governed opposite catalytic performance for C_3H_6 and C_3H_8 combustion: the effect of the Pt electronic structure and chemisorbed oxygen species. *Environ. Sci. Technol.* **55**, 9243–9254 (2021).

[4] Setvin, M.et al. Reaction of O_2 with subsurface oxygen vacancies on TiO_2 anatase (101). *Science* **341**, 988−991 (2013).

[5] Dong, J. et al. Insights into the CeO_2 facet-depended performance of propane oxidation over Pt-CeO_2 catalysts. *J. Catal.* **407**, 174-185 (2022).

[6] Bi, F. et al. Chlorine-coordinated Pd single atom enhanced the chlorine resistance for volatile organic compound degradation: mechanism study. *Environ. Sci. Technol.* **56**, 23, 17321-17330 (2022).

[7] Luo, Y. et al. Good interaction between well dispersed Pt and $LaCoO_3$ nanorods achieved Rapid Co^{3+}/Co^{2+} redox cycle for total propane oxidation. *Chem. Eng. J.* **357**, 395-403 (2019).

[8] Huang, Z. et al. Highly efficient oxidation of propane at low temperature over a Pt-Based catalyst by optimization support. *Environ. Sci. Technol.* **56**, 23, 17278-17287 (2022).
